# HIF1α promotes prostate cancer progression by increasing ATG5 expression

**DOI:** 10.1080/19768354.2019.1658637

**Published:** 2019-08-28

**Authors:** Kaiyuan Yu, Luxia Xiang, Shaoxun Li, Shuaibin Wang, Chaohao Chen, Haiqi Mu

**Affiliations:** a The Second Affiliated Hospital & YuYing Children’s Hospital of Wenzhou Medical University, Wenzhou City, People’s Republic of China; b The Second School of Medicine, Wenzhou Medical University, Wenzhou, People’s Republic of China

**Keywords:** HIF1α, ATG5, autophagy, PC-3 cell

## Abstract

Prostate cancer (PCa) is the most frequently diagnosed cancer among men. However, the major modifiable risk factors for PCa are poorly known and its specific mechanism of progression remains unclear. Here we reported that, in prostate cancer cells, the autophagy level was elevated under hypoxic condition, as well as the mRNA and protein level of ATG5, which is an important gene related to autophagy. Furthermore, we found HIF1α could directly bind to the promoter of ATG5 and promote the expression of ATG5 on transcriptional level by luciferase assay and ChIP assay. Intriguingly, overexpression of HIF1α by HIF1α-M could increase tumor size and the effect could be abolished by knockdown ATG5 by si-ATG5 in BALB/cA-nu/nu nude mice. Importantly, HIF1α could also promote the metastasis of PC-3 cells by upregulating the ATG5 and autophagy level and knockdown ATG5 and inhibition autophagy both could abolish the effect of overexpression of HIF1α on the migration of PC-3 cells. Taken together, our results, for the first time, proved that HIF1α could promote the proliferation and migration of PC-3 cells by direct upregulating ATG5 and autophagy level in PC-3 prostate cancer cells. Our findings not only provide new perspective for the relationship between hypoxia and autophagy, but also add new potential therapeutic regimens for the treatment of prostate cancers.

## Introduction

Today, prostate cancer (PCa) is the fifth most common cancer in the world and the most diagnosed cancer among men. (Torre et al. 2015). With the improvement of diagnosis and treatment, the mortality of PCa was decreasing over the years. However, the advanced malignancies were still refractory and might result in castration-resistant PCa and distant metastasis (Walczak and Carducci 2007; Draisma et al. 2009; Freedland 2011; Center et al. 2012; Cuzick et al. 2014). It has been reported that the development, proliferation and metastasis were not only regulated by androgen, but also associated with more than 80 genetic variants (Attard et al. 2011; Ishak and Giri 2011). However, the major modifiable risk factors for PCa were poorly known and its specific mechanisms still warrant further study.

Many studies have demonstrated that hypoxia plays important roles in tumor progression (Bertout et al. 2008). Deprivation of O_2_ might induce DNA replication, angiogenesis, metastatic potential in tumor tissues (van den Brenk et al. 1972; Young et al. 1988; Young and Hill 1990). The Hypoxia Inducible Factor 1 α (HIF1α) is a transcription factor response to hypoxia in cell and tissues (Wang et al. 1995; Majmundar et al. 2010). HIF1α is usually expressed in tumors and has recently been shown to increase the clinical risk of prostate cancer (Foley et al. 2009; Doe et al. 2012). Meanwhile, attention has been paid on autophagy in various cancers as it might play a dual role in the progression of tumor (Doria et al. 2013). On one hand, as a homeostatic autoregulation pathway, autophagy plays a vital role in suppressing tumor progression by clearing away damaged organelles and proteins. On the other hand, it also plays an important role in promoting tumor progression by helping tumor cells survive under unfavorable circumstances (Ravikumar et al. 2010; Mathew and White 2011).

Recent studies have reported that HIF1α increased the expression of autophagy gene BNIP3 in PCa and another autophagy gene BNIP3L also has been found under the regulation of oxygen tension (Hao et al. 2011; Selth et al. 2012). Here, we explored the relationship between hypoxia and autophagy and further evaluated the regulative role of HIF1α on autophagy pathway in prostate cancer. Our study revealed a novel positive regulation function of HIF1α on ATG5, key player in autophagy, in the PCa system. Furthermore, we found HIF1α could direct bind to the promoter of ATG5 and promote the expression of ATG5 on transcriptional level by luciferase assay and ChIP assay. Overexpression of HIF1α in PC-3 prostate cancer cells xenografts could increase tumor size and upregulate ATG5 expression. Importantly, HIF1α could also promote the metastasis of PC-3 cells by upregulating the ATG5 and autophagy level and konckdown ATG5 and inhibition autophagy both could abolish the effect of overexpression of HIF1α on the migration of PC-3 cells.

Taken together, our results proved that HIF1α could promote the proliferation and migration of PC-3 cells by direct upregulating ATG5 and autophagy level in PC-3 prostate cancer cells. Our findings not only provide a new perspective for the hypoxia and autophagy, but also add new potential therapeutic regimens for treatment of prostate cancers.

## Methods & materials

### Cell lines, plasmids and reagent

Human prostate cancer cell line PC-3 cell line was cultured in RIPM 1640 medium containing 10% fetal bovine serum (FBS), 100 unit/mL penicillin, and 100 mg/mL streptomycin. The cell was original obtained from the Cell Bank of Type Culture Collection of Chinese Academy of Sciences in Shanghai, China. For hypoxic condition, cells were cultured in a hypoxia incubator with 1% O_2_, 5% CO_2_ and 94% N_2_. Transfection was performing by using Lipo3000 (Invitrogen) according to instrunctions., ATG5(24922) and HIF1α-M(87261) were from addgene. HIF1α-M could continuously express HIF1α containing P402A/P564A/N803A mutation, so it could not be rapidly degraded under normoxic conditions. Si-HIF1α and Si-ATG5 oligos were purchased from Genepharma in Shanghai.

### RT-PCR & western blot

Total RNA were extracted from cells using Trizol reagent (Invitrogen), 1 μg RNA was reverse-transcribed into cDNA using the Prime Script RT (Takara). QRT-PCR was performed using Sybr Green (Takara) with a 7500 Real-Time PCR system (Applied Biosystems). Gene expression was normalized to 18S.

Protein was extracted using RIPA lysis buffer (50 mM Tris-HCl, pH 7.5, 150 mM NaCl, 1.0 mM EDTA, 0.1% SDS, and 1% Triton X-100). Protein lysates were separated by SDS-PAGE and subsequently transferred on to PVDF membranes using standard procedures. Primary antibody against HIF1α (BD), ATG5 (CST), LC3 (CST), P62 (BD) and Tubulin (Sigma Aldrich) were used.

### Luciferase assay

Dual Luciferase Assay Kit (Promega) was used according to the manufacturer’s instructions. Wild type ATG5 promoter was amplified by using human genomic DNA and subcloned into pGL3 plasmid. To generate HRE deletion ATG5 promoter, KOD-Plus mutagenesis kit (Toyobo) was used according to the manufacturer’s instruction. Then the pGL3 plasmids (WT or HRE deletion) were co-transfected with PRL-TK plasmids into PC-3 cells by using Lipofectamine 3000 (Invitrogen). Cells were exposed to normoxic or hypoxic condition for 48 h, then luciferase activity was measured.

### Chromatin immunoprecipitation assay

Chromatin immunoprecipitation assay was performed as described previously by using Magna ChIP™ Kit (Millipore). Briefly, PC-3 cells were exposed to hypoxic condition for 24 h, then chromatin was immunoprecipitated using anti-HIF1α antibody (Abcam). RT-PCR and PCR were performed to determine HIF1α binding site in ATG5 promoter.

### *In vivo* xenograft growth assay

HIF1α-M or vector transfected PC-3 cells (5 × 104) were suspended in 100 μl RIPM 1640 medium and subcutaneously injected into either side of the flank of the same male BALB/cA-nu/nu nude mice at 4-week-old. After 4 weeks, the mice were sacrified then the tumor tissue were frozen in lipid nitrogen for analysis.

### Migration assay and wound-healing assay

For the prostate cells migration assay, cells were transfected with lentivirus and siRNA oligos for 24 h, then 5 × 10^3^ to 10^4^ cells were maintained on the top of transwell chamber (Corning). PC-3 cells were seeded in 1640 medium without FBS in the top chamber. Cells were incubated for an additional 24 h then harvested for crystal violet staining and counting.

Wound-healing assay was performed according to the previous study (Rasheed et al. 2013). In brief, cells transfected with lentivirus and siRNA oligos for 24 h, then a wound was created in a cell monolayer. After 24 h, the migration of the cell was detected.

### Statistics analysis

All statistical analysis was expressed as mean ± SEM. Statistical analysis was conducted using Student *t*-test. *P* < 0.05 were considered to be significant.

## Results

### ATG5 is a hypoxia-responsive gene

**Figure 1.**
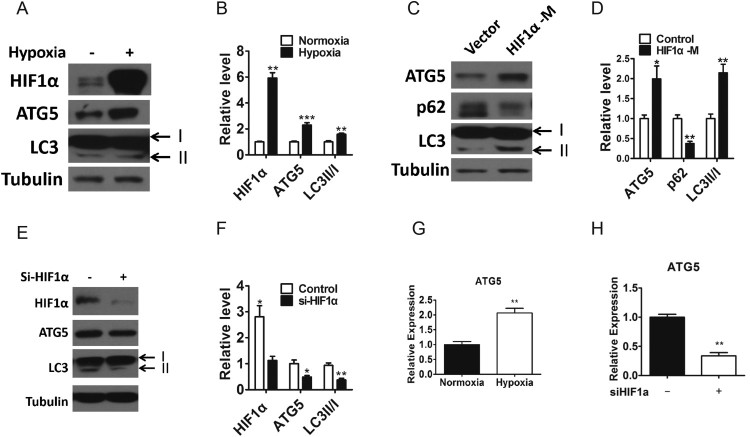
ATG5 is a hypoxia responsive gene. A–B, Western blotting(A) and densitometry(B) analysis of the protein levels of HIF1α, ATG5 and LC3 in PC-3 cells exposed to normoxia or 1% O_2_ for 24 h. Tubulin was used as a loading control. C–D, Western blotting (C) and densitometry (D) analysis of the protein levels of ATG5, p62 and LC3 in PC-3 cells transfected the HIF1α mutant plasmids (HIF1α-M) or vector for 48 h. Tubulin was used as a loading control. E–F, Western blotting (E) and densitometry (F) analysis of the protein levels of HIF1α, ATG5 and LC3 in PC-3 cells transfected with HIF1α siRNA (si-HIF1α) or nonsense control for 48 h. Tubulin was used as a loading control. G, Relative mRNA level of ATG5 in PC-3 cells exposed to normaxia or hypoxia for 24 h. H, Relative mRNA level of ATG5 in PC-3 cells transfected with HIF1α siRNA or nonsense control for 48 h. Means ± s.e.m are shown.*P < 0.05, **P < 0.01 and ***P < 0.001 (Student’s *t*-test). All experiments repeated at least twice and representative results are shown.

To assess the potential role of HIF1α on autophagy pathway in the prostate cancer, we first examined the protein levels of autophagy markers under hypoxic culture condition. PC-3 cells were exposed to normoxia (95% air, 5% CO_2_) or hypoxia (1% O_2_, 5% CO_2_, 94% N_2_) for 24 h. As expected, hypoxia can significantly increase the expression level of HIF1α, and we also found that the levels of ATG5 and LC3II/I, which are important molecules and markers in autophagy pathway, also increased significantly (Figure 1(A–B)), indicating that hypoxia may promote autophagy. According to above results, we sepporsed that hypoxia-responsive ATG5 may be regulated by HIF1α. Therefore, we overexpressed HIF1α in PC-3 cells by using HIF1α mutant (HIF1α-M) plasmids, and similarly, we found significantly increased in autophagy markers ATG5 and LC3II/I and significantly decreased in signaling protein p62 (Figure 1(C–D)) (Pankiv et al. 2007). Consistently, the protein levels of ATG5 and LC3II/I were significantly reduced after we silenced HIF1α in PC-3 cells by transfected with HIF1α-siRNA (si-HIF1α) (Figure 1(E–F)). We then determined the mRNA expression levels of ATG5 in the PC-3 cells under hypoxia or transfected with siHIF1α. Consistently, the relative expression of ATG5 was increased in PC-3 cell under hypoxia (Figure 1(G)) and decreased in the PC-3 cells transfected with siHIF1α (Figure 1(H)). These results suggest that hypoxia and HIF1α could promote the autophagy level and ATG5 might be regulated by HIF1α.

### HIF1α could regulate ATG5 by direct binding to the promoter of ATG5

**Figure 2.**
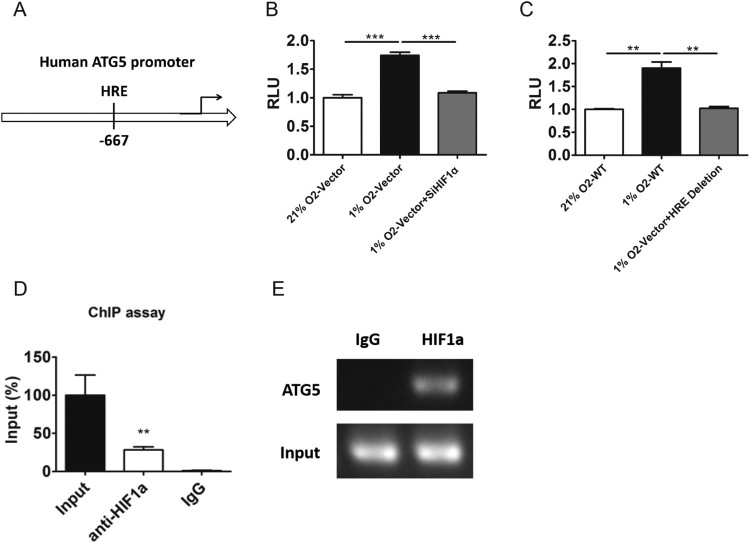
HIF1α could regulate ATG5 by direct binding to the promoter of ATG5. A, The schematic view of ATG5 genomic structure. The location of HIF1α responsive element (HRE) was shown. B, The transcriptional activity of reporter contain ATG5’s promoter were analyzed in PC-3 cells transfected with nonsense control or si-HIF1αexposed to normoxia or 1% O_2_ for 24 h. C. The transcriptional activity of reporter contain wild type or HRE deletion ATG5’s promoter were analyzed in PC-3 cells exposed to normoxia or 1% O_2_ for 24 h. D–E, ChIP analysis of ATG5 promoter was performed by using anti-HIF1α antibody in PC-3 cells exposed to 1% O_2_ for 24 h. RT-PCR (D) and PCR (E) were performed with primers specific to the functional HRE in ATG5 promoter. Means ± s.e.m are shown.*P < 0.05, **P < 0.01 and ***P < 0.001 (Student’s *t*-test). All experiments repeated at least twice and representative results are shown.

As is well known that, under hypoxic condition, HIF1α complex could bind to the HRE sites of the target gene’s promoter and induce the transcriptional activities of target genes (Binley et al. 2003; Wenger et al. 2005). Therefore, we hypothesized that HIF1α may regulate the expression of the ATG5 gene by binding to the promoter region of ATG5. As shown in Figure 2(A), we found that at least one hypoxia-response element (HRE) sequence located at the regions −667 in the upstream region of ATG promoter by online data analysis. To further verify our conjecture, the pGL3 luciferase reporter plasmids containing ATG5 promoter fragments along with pRL-TK plasmids were co-transfected into PC-3 cells, and these cells were exposed to normoxic or hypoxic conditions. Our data showed that the transcriptional activity of ATG5 promoter was significantly increased or decreased under hypoxic condition or both hypoxic and knockdown of HIF1α(Figure 2(B)). Consistently, deletion the HRE of the promoter of ATG5 could also abolish the effect of hypoxia on the activity of the reporter (Figure 2(C)). ChIP assay analysis showed that the degree of pull-down of chromatin fragments containing HRE in the ATG5 promoter by HIF1α was significantly higher than that of negative IgG control in PC-3 cells (Figure 2(D–E)). Those results indicate that the HIF1α can direct bind to the HRE of promoter of ATG5 and regulate the expression of ATG5 on the transcriptional level in PC-3 cells.

**Figure 3.**
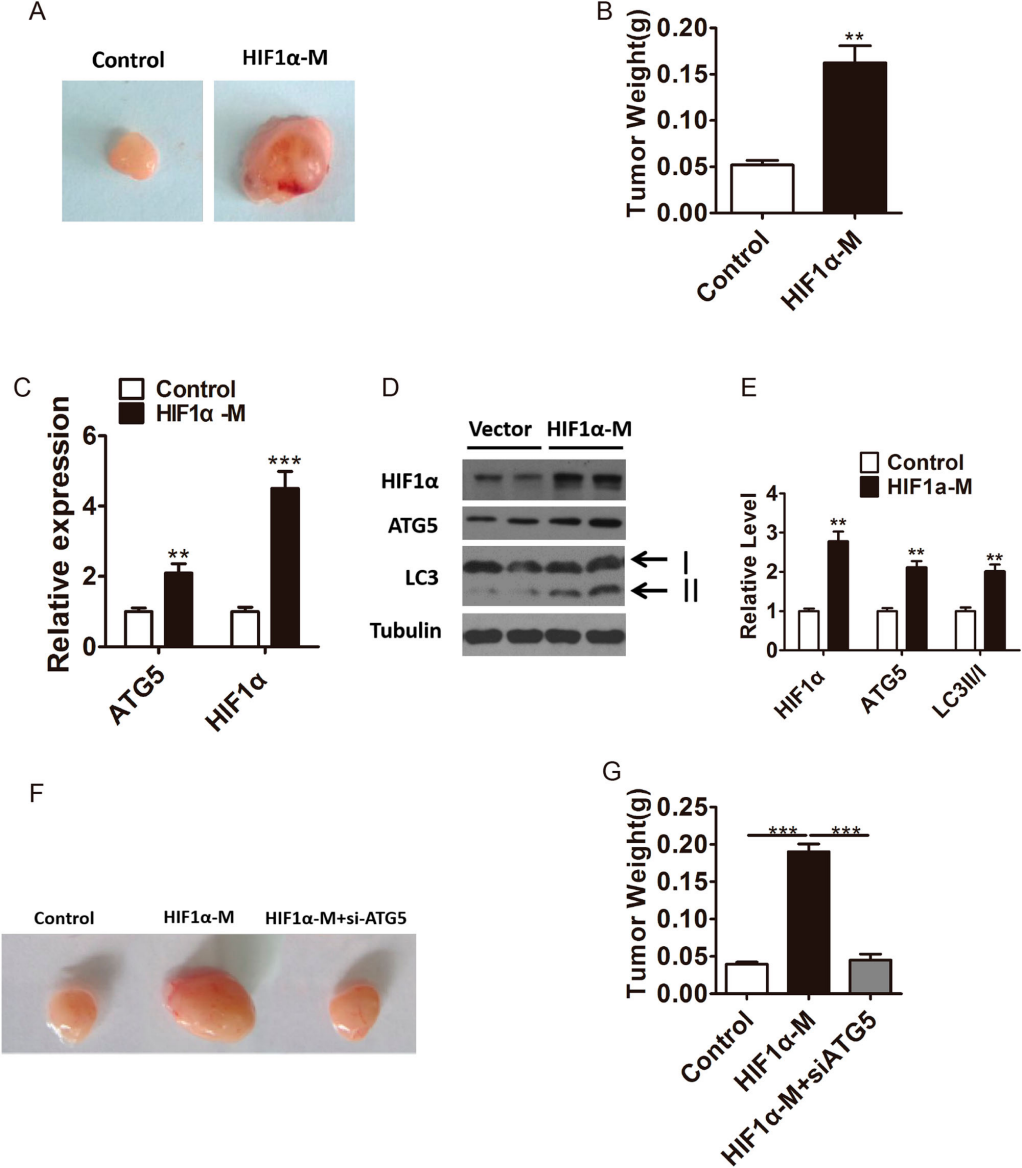
HIF1α promotes tumor cell proliferation *in vivo* by promoting ATG5 expression and autophagy levels. A–B, Representative images (A) and tumor weight (B) of tumor xenograft samples in nude mouses inoculated with wild type or HIF1α-overexpressd PC cells. C, Relative mRNA expression of HIF1α and ATG5 in harvested tumor xenograft samples of control or HIF1α-overexpressd group. D–E Western blotting (D) and densitometry (E) analysis of the protein levels of HIF1α, ATG5 and LC3 in harvested tumor xenograft samples of control or HIF1α-overexpressed group. Tubulin was used as a loading control. F–G, Representative images (F) and tumor weight (G) of tumor xenograft samples in nude mouses inoculated with wild type, HIF1α-overexpressd or both HIF1α-overexpressd and ATG5 konckdown PC cells Means ± s.e.m are shown. *P < 0.05, **P < 0.01 and ***P < 0.001 (Student’s *t*-test). All experiments repeated at least twice and representative results are shown.

HIF1α promotes tumor cell proliferation *in vivo* by promoting ATG5 expression and autophagy levels. To further assess the HIF1α’s regulation on ATG5 *in vivo*, we infected PC-3 cells with HIF1α-M lentivirus and then injected to BALB/cA-nu/nu nude mice. At the experiment endpoint, the HIF1α-overexpressed group showed significantly bigger tumor size compared to the control group (Figure 3(A–B)). Moreover, we examined the transcription levels in harvested tumor xenograft samples, and results showed that the mRNA levels of HIF1α and ATG5 were notably upregulated in HIF1α-overexpressed group (Figure 3(C)). Consistently, the protein levels of HIF1α, ATG5 and LC3II/I were also significantly increased after the upregulated of HIF1α (Figure 3(D–E)), indicating that HIF1α might promote the proliferation by inducing autophagy *in vivo*. Intriguingly, we found that knockdown of ATG5 could abolish the effect of HIF1α-overexpressed on the proliferation of PC-3 cells *in vivo* (Figure 3(F–G)). Taken together, our results indicated that HIF1α could promote tumor cell proliferation *in vivo* by promoting ATG5 expression and autophagy levels.

**Figure 4.**
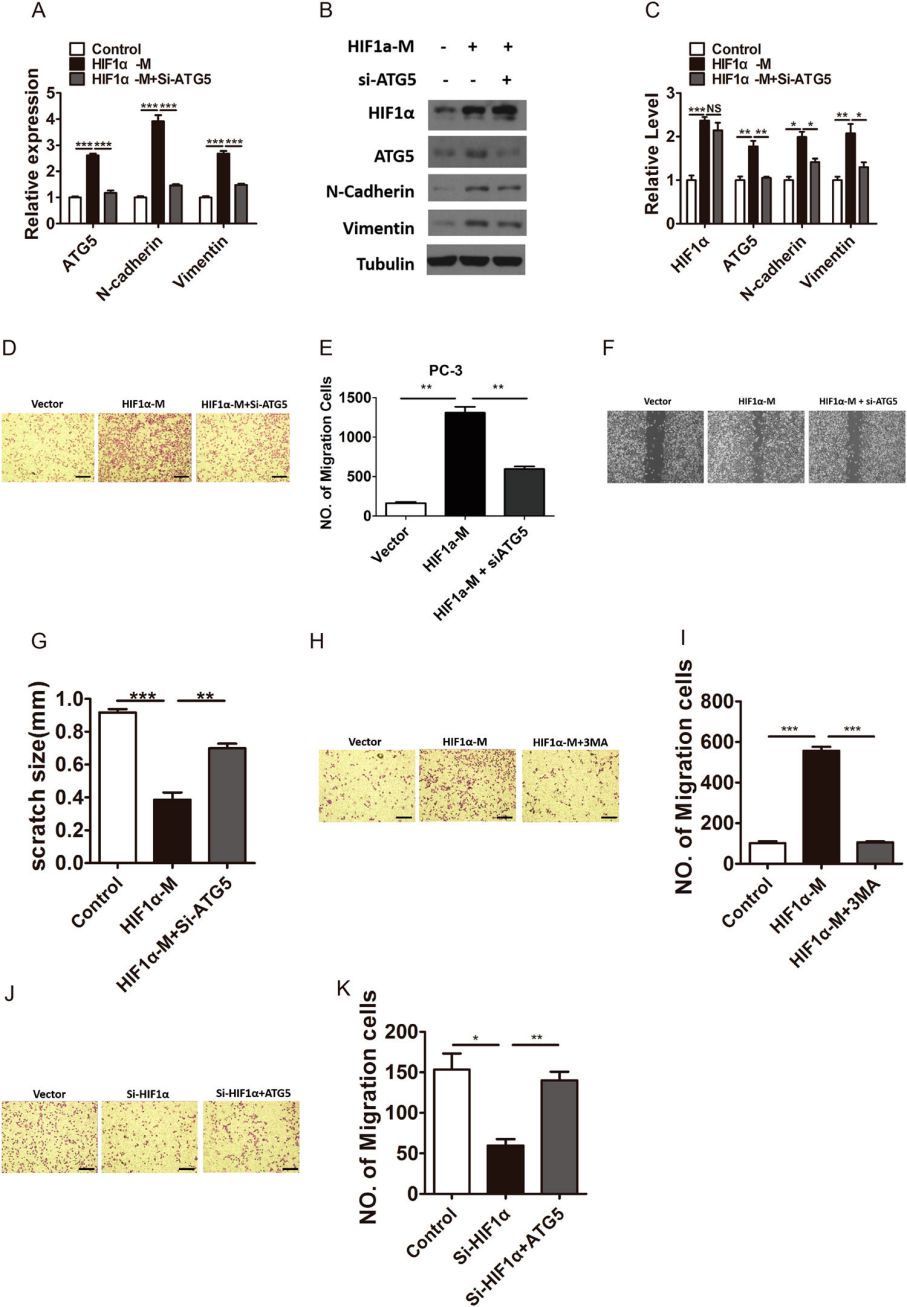
HIF1α promotes metastasis of PC-3 cells by promoting ATG5 expression and autophagy levels. A, Relative mRNA expression of ATG5, N-cadherin and Vimentin in PC-3 cells transfected with vector, HIF1α-M or both HIF1α-M and si-ATG5 oligonucleotide. B–C, Western blotting (B) and densitometry (C) analysis of the protein levels of HIF1α, ATG5, N-cadherin and Vimentin in PC-3 cells transfected with vector, HIF1α-M or both HIF1α-M and si-ATG5 oligonucleotide. Tubulin was used as a loading control. D–E, Representative photographs (D) and number of migration cells (E) of transwell migration assay of PC-3 cells transfected with Vector, HIF1α-M or both HIF1α-M and si-ATG5 oligonucleotide. Scale bar, 200 uM.HIF1αHIF1αHIF1αHIF1αF-G, Representative photographs (F) and quantification of scratch size (G) of wound-healing assay of PC-3 cells transfected with Vector, HIF1α-M or both HIF1α-M and si-ATG5 oligonucleotide.HIF1αHIF1α. H-I, Representative photographs (H) and number of migration cells (I) of transwell migration assay of PC-3 cells treated with Vector, HIF1α-M or both HIF1α-M and 3MA. Scale bar, 200 uM. J-K, Representative photographs (J) and number of migration cells (K) of transwell migration assay of PC-3 cells transfected with Vector, si-HIF1α or both si-HIF1α-M and ATG5 exposed to hypoxia. Scale bar, 200 uM. Means ± s.e.m are shown.*P < 0.05, **P < 0.01 and ***P < 0.001(Student’s *t*-test). All experiments repeated at least twice and representative results are shown.

HIF1α promotes metastasis of PC-3 cells by promoting ATG5 expression and autophagy levels based on the above results, we wanted to investigate whether the progression of prostate tumors can be prevented by inhibiting the expression level of ATG5. We next silenced ATG5 with si-ATG5 in PC-3 cells after overexpression of HIF1α, and we found that the mRNA and protein levels of N-cadherin and Vimentin were significantly decreased after the inhibition of ATG5 (Figure 4(A–C)). Since N-cadherin and Vimentin have been proved usually positive in various cancer metastases (Ivaska et al. 2007; Repetto et al. 2014), we speculated the downregulation of ATG5 might impact the HIF1α-induced metastasis ability of PC-3 cells. We then found that the hypoxia can significantly promote the invasion of PC-3 cell, at the same time, the invasion number of PC-3 cell was significantly decreased after the inhibition of ATG5 by si-ATG5 (Figure 4(D–E)). Furthermore, wound-healing assay has also been performed to test the effect of ATG5 on the metastasis of PC-3 cells. Consistently, normal wound-healing process was dysregulated in PC-3 cells transfected with HIF1α-M that serious metastatic spread was observed. However, in PC-3 cells transfected with both HIF1α-M and si-ATG5, metastatic spread was significantly reduced despite that the HIF1α-overexpressed microenvironment was considered ‘suitable’ for metastasis (Figure 4(F–G)). To further investigate the relationship between HIF1α and autophagy in the migration of PC-3 cells, we inhibited autophagy level with 3MA, a well-known autophagy inhibitor, after overexpression of HIF1α. We found that inhibition of autophagy can significantly inhibit the invasion of PC-3 cells (Figure 4(H–I)). Consistently, knockdown HIF1α by si- HIF1α could significantly decrease the invasion cell number and overexpression of ATG5 could rescue the invasion ability of PC-3 cells (Figure 4(J–K)). All these above results indicating that HIF1α promotes metastasis of PC-3 cells by promoting ATG5 expression and autophagy levels.

## Discussion

As one of the carcinomata in the male reproductive system, PCa poses severe health threats to men especially the elderly, yet there is no cure therapy for advanced situation till now. Extensive clinical studies pointed out that genetic variants of autophagy pathway contribute to the variable patient outcomes so that autophagy proteins might become novel prognostic markers for PCa (Giatromanolaki et al. 2014; Huang et al. 2015). Autophagy is a homeostatic stress response that reacts under environmental stimulus to maintain homeostasis by eliminating unnecessary cytoplasmic components (Seglen and Bohley 1992; Lum et al. 2005). In normal cells, autophagy is generally beneficial, and facilitates to eliminate excessive proteins and organelles and promote cell survival (Lian et al. 2011). However, in pathological conditions, like the prostate cancer system, the role of autophagy is much more complicated and multi-faceted (Levine and Kroemer 2008). Some studies demonstrated a tumor-suppressive function of autophagy in PCa: induction of autophagy by (−)-gossypol could trigger the autophagic cascade, promoting the killing of cancer cells (Lian et al. 2011); additionally autophagy-defection caused by high levels autophagy inhibitors predicts poor prognosis of PCa patients (Jiang et al. 2014; Jiang et al. 2015). On the other hand, some reports emphasized a tumor accelerative function of autophagy; like autophagy might inhibit apoptosis and promote a regenerative metabolism in PC cells (Herman-Antosiewicz et al. 2006; Hahm et al. 2014).

Hypoxia happens to be exactly one of the typical environmental stress. With high expression of a hypoxia, serial autophagy markers, such as BNIP3, LC3B and ATG5, were found modulated by HIF1α, and these were often associated with aggressive tumor progression phenotype *in vivo* (Giatromanolaki et al. 2004; Rouschop et al. 2010). In the present study, we emphasized the protein value of autophagy marker proteins in both the presence and absence of HIF1α. We found in PC cells, hypoxia could lead to increased expression of autophagy markers ATG5 and LC3 but downregulation of p62. Since it’s known that, during autophagy, isolated membranes elongate to form a cup-shaped phagophore and then the phagophore gradually enclose cytoplasmic cargo, generating autophagosomes (Weidberg, Shvets, et al. 2011; Kraft and Martens 2012; Rubinsztein et al. 2012). This process includes ubiquitinated two conjugation systems, the ATG5-ATG12 conjugate and LC3-ATG8 conjugate, which are essential for the autophagosome formation (Hanada et al. 2007; Weidberg, Shpilka, et al. 2011). Besides, the degradation of cargo recruiter-p62 has also been reported to be associated with the autophagosomes formation (Clausen et al. 2010). Hence, our results fully explain that the overexpression of HIF1α upregulates autophagy proteins and hypoxic treatment could promote the autophagosomes formation and induce autophagy in PCa system. Subsequently, via online bioinformatic analysis, we discovered a potential HIF1α-HRE binding site on the −667 of ATG5 promoter region. By qPCR ananlysis, luciferase activity measurement and ChIP assay, we verified the putative binding site and proved that hypoxia or HIF1α enhance ATG5 expression in transcriptional levels through direct bind to the HRE site of AGT5 promoter.

On the other hand, we also delved into the complementary relationship between ATG5 and HIF1α in PCa. Our data proved that the silence of ATG5 under hypoxia condition could downregulate the expression of epithelial–mesenchymal transition markers N-cadherin and Vimentin, which can induce malignant transformation in cell carcinomas (Rodriguez et al. 2013; Zhang et al. 2014). This is interesting because as we all know that hypoxic microenvironment was considered to be suitable for malignant transformation or tumor cell migration. Studies suggest that activation of HIF signaling might influence effective wound-healing processes and cause the influx of angiogenic cytokines from nearby immune cells that can stir up metastatic spread (Finger and Giaccia 2010; Langley and Fidler 2011), nevertheless adaptation to hypoxic stress via HIF signaling could also promote wound-healing processes (Andrikopoulou et al. 2011; Hong et al. 2014). Our results proved that silence of ATG5 enhanced the positive wound healing and inhibited metastatic spread of tumor cells, finally indicating downregulation of ATG5 alleviates HIF1α-induced metastasis in PCa.

Here, our data provide experimental evidence to show, activation of autophagy in PCa under the hypoxic condition. These results suggest that the autophagy progress in prostate cancer is directly proportional with hypoxia. As is confirmed that HIF1α commonly overexpresses in pejorative PCa, it’s reasonable for us to suspect that HIF1α might deteriorate PCa by accelerating autophagy and upregulating ATG5 to promote the cancer cell proliferation and migration in PCa. Further studies of the relevant pathways of HIF1α in promoting PCa cells autophagy and its associated mechanisms with PCa development and progression may uncover new mechanisms for PCa progression and therapeutic intervention.
